# Study on the application of serological indicators combined with ultrasonic cardiogram in predicting recurrence of radiofrequency ablation in atrial fibrillation patients

**DOI:** 10.1097/MD.0000000000047014

**Published:** 2026-03-13

**Authors:** Jia-Fa Jin, Shen Huang, Kun Feng, Xiu-Yu Wang

**Affiliations:** a Department of Cardiology, Affiliated Hospital of Chengdu University, Chengdu, Sichuan, China.

**Keywords:** atrial fibrillation, N-terminal brain natriuretic peptide precursor, prediction, recurrence, troponin I, ultrasonic cardiogram

## Abstract

Atrial fibrillation (AF) is associated with high rates of recurrence after radiofrequency ablation (RFA). This retrospective observational study was conducted in accordance with the Strengthening the Reporting of Observational Studies in Epidemiology statement to evaluate whether combined serological and echocardiographic indicators predict postablation recurrence. We retrospectively reviewed 97 nonvalvular AF patients who underwent 1st-time RFA at our institution between April 2021 and April 2023. Patients were classified into recurrence (n = 21) and nonrecurrence (n = 76) groups based on documented AF recurrence within 12 months. Data collection followed prespecified variable definitions: demographic and clinical characteristics, serological markers (N-terminal pro-B-type natriuretic peptide [NT-proBNP] and cardiac troponin I [cTnI]), and standardized echocardiographic parameters (left atrial diameter [LAD], left ventricular end-diastolic diameter [LVDD], and left ventricular ejection fraction [LVEF]). We controlled for selection bias by applying strict inclusion/exclusion criteria and blinded measurement of all variables. Logistic regression identified independent predictors of recurrence, and receiver operating characteristic curve analysis quantified predictive performance. After adjustment for confounders, elevated NT-proBNP, cTnI, LAD, and LVDD were independent risk factors (OR range 2.401–3.251; *P* < .05), while higher LVEF was protective (OR = 0.279; *P* < .05). The combined model achieved an area under the curve of 0.771 (95% CI: 0.660–0.883; sensitivity 74.1%, specificity 75.7%). In this retrospective study, the combined measurement of NT-proBNP, cTnI, LAD, LVDD, and LVEF demonstrated robust discriminatory power for predicting AF recurrence after RFA.

## 1. Introduction

Atrial fibrillation (AF) stands as one of the most prevalent continuous arrhythmias in clinical practice, significantly contributing to stroke, cardiovascular diseases, and mortality.^[1,2]^ With the annual increase in the incidence of cardiovascular and cerebrovascular diseases, the prevalence of AF is also on the rise. Recent studies have reported the prevalence of around 33.5 million cases of AF, culminating in substantial increases in healthcare costs and mortality rates.^[3]^ Despite substantial time and effort invested in understanding the pathophysiology of AF and developing improved treatment strategies, it remains challenging to pinpoint the underlying mechanisms in individual AF patients, and the therapeutic outcomes are often suboptimal. This underscores the urgent need for novel therapeutic approaches to enhance the current treatment landscape.^[4,5]^ Presently, common clinical strategies for managing AF encompass lifestyle modifications, medication control, nutritional therapy, substrate-based ablation, and neuroregulation, all of which help maintain sinus rhythm within patients.^[6]^ Although radiofrequency ablation (RFA) has become increasingly refined in treating AF, studies have denoted that the postprocedure recurrence rate remains high, necessitating repeated ablation for a considerable number of patients.^[7]^ Therefore, addressing the issue of AF recurrence subsequent to RFA is still a crucial topic in current clinical research.

In recent years, the value of the serological markers N-terminal pro-B-type natriuretic peptide (NT-proBNP) precursor and cardiac troponin I (cTnI) in the diagnosis and prognosis of cardiovascular diseases has been widely recognized. Studies have confirmed that serum levels of NT-proBNP and cTnI remarkably increase in individuals with heart failure, myocardial injury, and cardiac insufficiency. The changes in these levels are highly correlated with the occurrence of adverse cardiovascular events, making them effective and sensitive parameters for forecasting cardiovascular diseases.^[8,9]^ Ultrasonic cardiogram (UCG) is adopted to identify damage to the heart and injury of large blood vessels, assess myocardial function, and provide real-time observation of cardiac systolic and diastolic functions. It offers quantitative or semiquantitative diagnosis for detecting blood flow velocity. This technique is valuable for diagnosing and distinguishing conditions such as cardiomyopathy, congenital heart disease, pericarditis, coronary atherosclerotic cardiopathy, aortic aneurysm, and valvular heart diseases.^[10,11]^ Additionally, recent studies have corroborated its effectiveness in monitoring AF.^[12]^

Based on this, our current study focuses on AF patients undergoing RFA at our medical facility to probe the combined value of serum NT-proBNP and cTnI with UCG in forecasting postoperative recurrence. We aim to contrast the predictive efficacy of these individual and combined indicators for postoperative recurrence to identify a more reasonable and efficacious approach for predicting recurrence in AF patients, thereby enhancing their prognosis.

## 2. Study materials and methods

### 2.1. Selection of subjects

This single-center, retrospective observational study was conducted at the Department of Cardiology, Affiliated Hospital of Chengdu University, Chengdu, China. From April 2021 to April 2023, 97 consecutive nonvalvular AF patients underwent 1st-time RFA at our institution; all met the predefined inclusion and exclusion criteria and were therefore included in the analysis. Reporting follows the Strengthening the Reporting of Observational Studies in Epidemiology checklist. The 97 patients were allocated into the recurrence group (n = 21) and the nonrecurrence group (n = 76) as per whether they underwent recurrence within the year postprocedure. Within the recurrence group, there were 12 males and 9 females; the age range was 36 to 80 years, with an average age of (58.22 ± 5.46) years; the body mass index (BMI) ranged from 19 to 27 kg/m^2^, with an average BMI of (21.68 ± 3.77) kg/m^2^. The nonrecurrence cohort consisted of 41 males and 35 females with an age range from 35 to 80 years, an average age of (59.05 ± 5.19) years, a BMI ranging from 19 to 28 kg/m^2^, as well as an average BMI of (22.03 ± 2.94) kg/m^2^.

### 2.2. Participants and sample selection

We identified all consecutive nonvalvular AF patients aged ≥ 18 years undergoing 1st-time RFA in the study period.

Inclusion criteria: Paroxysmal or persistent AF; continuous electrocardiogram (ECG) monitoring during hospitalization; 1st RFA; complete pre and postoperative data.

Exclusion criteria: Hepatic or renal dysfunction; psychiatric disorder; end-stage disease (life expectancy < 1 year); acute coronary events within 6 months; thyroid dysfunction; pregnancy or lactation.

### 2.3. Data collection and variable definitions

Data sources included electronic medical records and laboratory databases. Trained research staff extracted.

Demographics and comorbidities: Age, sex, BMI, hypertension, diabetes, cerebrovascular disease, AF type, New York Heart Association (NYHA) class.

Serological markers: NT-proBNP (pg/mL) and cTnI (ng/mL) measured on day 2 postadmission by chemiluminescence immunoassay (Wuhan Mingde Biotechnology Co., Ltd., Wuhan, China).

Echocardiographic parameters: Left atrial diameter (LAD) (mm), left ventricular end-diastolic diameter (LVDD) (mm), left ventricular ejection fraction (LVEF) (%) obtained by a GE LOGIQ E9 system (GE Healthcare, Chicago) with ML6-15 probe (3.5 MHz). Three cardiac cycles were recorded in the left lateral decubitus position; measurements were performed offline by an experienced sonographer blinded to clinical outcomes.

### 2.4. Examination of serological indicators

On the 2nd day postadmission, fasting venous blood samples were drawn from the patients elbows. The levels of NT-proBNP and cTnI were gauged using chemiluminescence immunoassay (kits purchased from Wuhan Mingde Biotechnology Co., Ltd.). All procedures were conducted strictly in accordance with the reagent instructions.

### 2.5. UCG examination

A LOGIQ E9 color Doppler ultrasound diagnostic device, manufactured by General Electric (GE), was used along with an ML6-15 probe at a frequency of 3.5 MHz. All subjects were positioned in the left lateral position and were in a resting state during the examination. Standard 2-dimensional ultrasonography was harnessed to harvest the forward flow spectrum of the mitral valve. In the 3-dimensional mode, QLAB analysis software (Philips Healthcare, Andover) was applied to place 5 marker points at the top of the left ventricle and left atrium, as well as the mitral annular. The software automatically generated a 3D image of the left heart, obtaining measurements for the LVDD, LAD, LVEF, and left ventricular mass index (LVMI) from the apical 4-chamber view.

Dynamic images for 3 consecutive cardiac cycles were captured and stored for offline analysis. All parameters were determined 3 times and averaged. The entire procedure was performed by the same ultrasound physician with 5 years of experience.

### 2.6. Postoperative management and follow-up

All patients received guideline-directed postablation care under the supervision of our electrophysiology team. Antiarrhythmic drug therapy – typically amiodarone or dronedarone – was prescribed for 3 months to reduce early recurrence, unless contraindicated. Anticoagulation (warfarin or direct oral anticoagulant) was continued for at least 3 months postRFA and thereafter based on individual CHA_2_DS_2_-VASc scores. Rate-control agents (β-blockers or calcium-channel blockers) were adjusted as needed to maintain resting heart rate < 80 bpm. Scheduled follow-up visits occurred at 3, 6, and 12 months, during which patients underwent 12-lead ECG and 24-hour Holter monitoring. Any symptomatic episodes were recorded with event monitors or emergency ECGs.

### 2.7. Statistical analysis

We 1st evaluated the extent of missing data and, for variables with <5% missingness, applied multiple imputation by chained equations in SPSS 22.0 to generate 5 imputed datasets and pooled estimates; variables with under 1% missingness were handled by listwise deletion. Continuous variables approximating normality are presented as mean ± standard deviation and compared by Student *t* test (with log-transformation for skewed data), while categorical variables are expressed as counts (percentages) and analyzed using the χ^2^ or Fisher exact test as appropriate. Univariate analyses identified candidate predictors of recurrence (variables with *P* < .10 and clinically established factors such as age, sex, BMI, and NYHA class), which were then entered simultaneously into a multivariate logistic regression model; continuous predictors (age, BMI, NT-proBNP, cTnI, LAD, LVDD, and LVEF) remained in their original form, while binary variables (sex and comorbidities) were coded as 1 = yes and 0 = no, and NYHA class was represented by dummy variables with Class I/II as the reference. We assessed multicollinearity via variance inflation factors (<2 for all predictors) and report adjusted odds ratios with 95% confidence intervals. Predictive performance of individual and combined models was evaluated using receiver operating characteristic (ROC) curve analysis (DeLong method) to calculate area under the curve (AUC) with 95% CI, and sensitivity and specificity were determined at the optimal Youden index. Model calibration was evaluated using the Hosmer–Lemeshow test and calibration plots, and internal validation was performed with 1000 bootstrap resamples. All tests were 2-tailed with α = 0.05, and analyses were performed in SPSS version 22.0 (IBM Corp., Armonk).

## 3. Results

### 3.1. Comparison of general data between the 2 groups

No statistically significant differences were noticed between the recurrence and nonrecurrence groups in terms of gender, age, BMI, medical history, and type of AF (*P* > .05). Within the recurrence group, there were 4 patients classified as NYHA class II, 6 as class III, and 11 as class IV. As for the nonrecurrence group, there were 25 patients classified as NYHA class II, 33 as class III, and 18 as class IV. The variance between the 2 groups was deemed to be statistically significant (*P* < .05), as displayed in Table 1. Our findings suggested that, apart from NYHA functional classification, there existed no disparities in baseline data between the 2 groups. The proportion of patients in NYHA class IV was heightened in the recurrence cohort as opposed to the nonrecurrence group. We speculated that a higher NYHA functional class might boost the likelihood of postoperative recurrence in AF patients.

**Table 1:** Comparison of general data between the 2 groups ($\bar{x}\pm \;s$, n).

Data		Recurrence group (n = 21)	Nonrecurrence group (n = 76)	*χ^2^*/*t*	*P*
Gender (cases)	Male	12 (57.14)	41 (53.95)	0.068	.765
	Female	9 (42.86)	35 (46.05)		
Age (years)	–	58.22 ± 5.46	59.05 ± 5.19	0.650	.517
BMI (kg/m^2^)	–	21.68 ± 3.77	22.03 ± 2.94	0.453	.652
Medical history (cases)	Hypertension	5 (23.81)	24 (31.58)	1.004	.605
	Diabetes	7 (33.33)	28 (36.84)		
	Cerebrovascular disease	9 (42.86)	24 (31.58)		
AF type (cases)	Paroxysmal AF	15 (71.43)	39 (51.32)	2.697	.101
	Persistent AF	6 (28.57)	37 (48.68)		
NYHA cardiac function classification (cases)	Class II	4 (19.05)	25 (32.89)	6.490	0.039
	Class III	6 (28.57)	33 (43.42)		
	Class IV	11 (52.38)	18 (23.68)		

AF = atrial fibrillation, BMI = body mass index, NYHA = New York Heart Association.

### 3.2. Comparison of serological indicators between the 2 groups

Within the recurrence cohort, serum levels of NT-proBNP and cTnI stood at (985.64 ± 74.29) pg/mL and (0.89 ± 0.07) ng/mL, respectively, which were higher than those within the nonrecurrence group ([755.41 ± 56.38) pg/mL and (0.26 ± 0.03] ng/mL, respectively), with statistically significant variances (*P* < .05), as detailed in Table 2. Our findings demonstrated that postoperative recurrence in AF patients undergoing RFA was associated with significantly elevated levels of the serum injury markers NT-proBNP and cTnI. Monitoring these levels might help forecast postoperative recurrence in patients.

**Table 2:** Comparison of serum NT-proBNP and cTnI levels between the 2 groups ($\bar{x}\pm \;s$).

Serological indicators	Recurrence group (n = 21)	Nonrecurrence group (n = 76)	*t*	*P*
NT-proBNP (pg/mL)	985.64 ± 74.29	755.41 ± 56.38	15.413	<.001
cTnI (ng/mL)	0.89 ± 0.07	0.26 ± 0.03	61.226	<.001

cTnI = cardiac troponin I, NT-proBNP = N-terminal pro-B-type natriuretic peptide.

### 3.3. Comparison of UCG parameters between the 2 groups

Within the recurrence group, the UCG parameters LAD, LVDD, LVEF, and LVMI reached (36.18 ± 4.87) mm, (44.87 ± 5.62) mm, (56.73% ± 6.11%), and (140.76 ± 18.90) g/m^2^, respectively. As opposed to the nonrecurrence cohort, there was no statistically significant disparity in LVMI levels between the 2 groups (*P* > .05). Notwithstanding, LAD and LVDD were dramatically elevated, while LVEF was significantly lower in the recurrence cohort vis-à-vis the nonrecurrence cohort, with statistically significant variances (*P* < .05). See Table 3. This demonstrated that patients with recurrent AF following RFA exhibited impaired left ventricular diastolic function, lowered LVEF, and more severe myocardial damage compared to individuals in the nonrecurrence group.

**Table 3:** Comparison of LAD, LVDD, LVEF, and LVMI levels between the 2 groups ($\bar{x}\pm \;s$).

Serological parameters	Recurrence group (n = 21)	Nonrecurrence group (n = 76)	*t*	*P*
LAD (mm)	36.18 ± 4.87	32.94 ± 4.02	3.119	.002
LVDD (mm)	44.87 ± 5.62	41.36 ± 4.55	2.969	.004
LVEF (%)	56.73 ± 6.11	63.21 ± 5.48	4.678	<.001
LVMI (g/m^2^)	140.76 ± 18.90	135.43 ± 16.74	1.256	.212

LAD = left atrial diameter, LVDD = left ventricular end-diastolic diameter, LVEF = left ventricular ejection fraction, LVMI = left ventricular mass index.

### 3.4. Analysis of relevant factors influencing postoperative recurrence in AF patients

We incorporated the statistically significant factors into a logistic multivariate regression analysis, and the results displayed that serum markers NT-proBNP and cTnI, along with UCG indicators LAD, LVDD, and LVEF, were all independent influencing factors for postoperative recurrence in AF individuals. High-level NT-proBNP, cTnI, LAD, and LVDD (OR = 2.787, 2.401, 3.251, 2.570) were identified as risk factors, whereas heightened LVEF (OR = 0.279) was identified as a protective factor (*P* < .05), as detailed in Table 4. This hinted that in a clinical setting, special attention should be given to alterations in serum markers NT-proBNP and cTnI, as well as UCG indicators LAD, LVDD, and LVEF, in patients undergoing RFA for AF, in order to minimize the risk of postoperative recurrence as much as possible.

**Table 4:** Analysis of relevant factors influencing postoperative recurrence in AF patients.

Factor	β	SE	Wald	OR	95% CI	*P*
NT-proBNP	1.025	0.431	5.656	2.787	1.198–6.487	.018
cTnI	0.876	0.332	6.962	2.401	1.253–4.603	.009
LAD	1.179	0.504	5.472	3.251	1.211–8.731	.020
LVDD	0.944	0.385	6.012	2.570	1.209–5.466	.015
LVEF	−1.278	0.615	4.361	0.279	0.084–0.925	.037

AF = atrial fibrillation, CI = confidence interval, cTnI = cardiac troponin I, LAD = left atrial diameter, LVDD = left ventricular end-diastolic diameter, LVEF = left ventricular ejection fraction, NT-proBNP = N-terminal pro-B-type natriuretic peptide.

### 3.5. The value of serum markers and UCG parameters in forecasting postoperative recurrence in the context of AF

**Figure 1.**
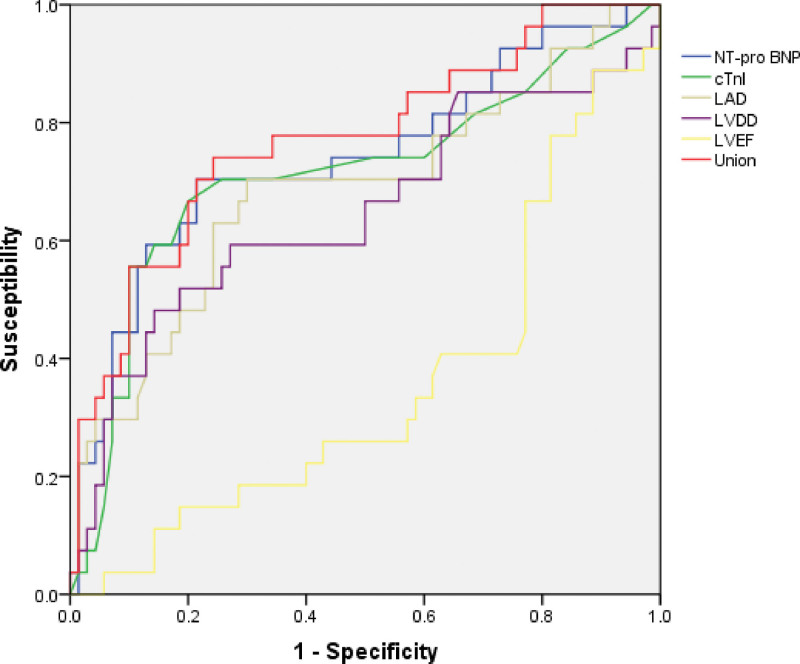
The value of serum markers, UCG parameters, and their combination in predicting postoperative recurrence of AF. AF = atrial fibrillation, cTnI = cardiac troponin I, LAD = left atrial diameter, LVDD = left ventricular end-diastolic diameter, LVEF = left ventricular ejection fraction, NT-proBNP = N-terminal pro-B-type natriuretic peptide, UCG = ultrasonic cardiogram.

ROC curve analysis denoted that the highest predictive efficiency for AF recurrence was achieved when NT-proBNP was above 807.17 pg/mL, cTnI was above 0.3 ng/mL, LAD was above 34.99 mm, LVDD surpassed 43.14 mm, and LVEF was ≤ 59.15%. The combined prediction of these 5 factors for AF recurrence exhibited an AUC of 0.771, with a sensitivity of 74.07% and specificity of 75.71% (95% CI: 0.660–0.883, *P* < .001), as displayed in Table 5 and Figure 1. Our findings confirmed that versus the single testing of serum markers and echocardiographic parameters, the combined testing of these 5 factors provided higher value in predicting and distinguishing postoperative recurrence in AF patients undergoing RFA.

**Table 5:** The value of serum markers, UCG parameters, and their combination in predicting postoperative recurrence of AF.

Parameter	Cutoff	AUC	Sensitivity (%)	Specificity (%)	Youden index	95% CI	*P*
NT-proBNP	>807.17 pg/mL	0.740	70.37	78.57	0.4894	0.618–0.862	<.001
cTnI	>0.3 ng/mL	0.713	66.67	80.00	0.4667	0.583–0.843	.001
LAD	>34.99 mm	0.687	70.37	70.00	0.4037	0.557–0.816	.005
LVDD	>43.14 mm	0.649	48.15	85.71	0.3386	0.511–0.787	.034
LVEF	≤59.15%	0.647	59.26	75.71	0.3497	0.523–0.772	.021
Combination of 5 indicators	–	0.771	74.07	75.71	0.4979	0.660–0.883	<.001

AF = atrial fibrillation, AUC = area under the curve, CI = confidence interval, cTnI = cardiac troponin I, LAD = left atrial diameter, LVDD = left ventricular end-diastolic diameter, LVEF = left ventricular ejection fraction, NT-proBNP = N-terminal pro-B-type natriuretic peptide, UCG = ultrasonic cardiogram.

In addition to identifying the optimal cutoff values by the Youden index, we further explored the sensitivity–specificity trade-offs at alternative thresholds to reflect clinical decision-making. For example, for NT-proBNP, lowering the cutoff to 750 pg/mL increased sensitivity to 85.2% but reduced specificity to 65.7%, whereas raising the cutoff to 850 pg/mL improved specificity to 82.9% at the expense of sensitivity (63.0%). Similar trade-offs were observed for other predictors (Table S1, Supplemental Digital Content, https://links.lww.com/MD/R545). These findings suggest that while the Youden index provides a statistically optimal cutoff, clinicians may choose different thresholds depending on whether higher sensitivity (minimizing missed high-risk patients) or higher specificity (avoiding overtreatment of low-risk patients) is prioritized.

### 3.6. Post hoc power analysis

A post hoc power analysis was performed for the 5 primary predictors identified in the multivariate logistic regression model: NT-proBNP, cTnI, LAD, LVDD, and LVEF. Based on observed effect sizes, a significance level of 0.05, and a target power of 80%, the calculated post hoc power values were > 0.999 for NT-proBNP and cTnI, reflecting extremely large effect sizes (Cohen *d* > 3.0, and in some comparisons > 10); >0.99 for LVEF, corresponding to a large effect size (*d* ≈ 1.1); and 0.86 and 0.84 for LAD and LVDD, respectively, consistent with moderate-to-large effect sizes (*d* ≈ 0.7). These results confirm that the study had near-complete statistical power for the most critical variables, with adequate sensitivity to detect meaningful differences across all predictors.

### 3.7. Missing data assessment

The overall extent of missing data in this study was minimal. Across the analyzed variables, missingness ranged from 0.0% to 4.3%, with demographic and comorbidity data < 1% and serological/echocardiographic parameters < 5%. Little missing completely at random test indicated that the missing values were consistent with a missing completely at random pattern (*P* = .291). In accordance with best practices, variables with < 5% missingness were addressed using multiple imputation by chained equations with 5 imputations and pooled estimates, while variables with <1% missingness were managed by listwise deletion. This approach minimized bias and preserved statistical validity without altering the overall results (Table S2, Supplemental Digital Content, https://links.lww.com/MD/R545).

### 3.8. Model validation and calibration

The logistic regression model demonstrated good calibration, as indicated by a nonsignificant Hosmer–Lemeshow goodness-of-fit test (χ^2^ = 7.42, *P* = .47). Calibration plots further showed close agreement between predicted and observed recurrence probabilities. Internal validation with 1000 bootstrap resamples yielded an optimism-corrected AUC of 0.768, which was consistent with the original model performance (AUC = 0.771), supporting the robustness and stability of the predictive model.

## 4. Discussion

With the increasing aging population in recent years, AF has become a significant social burden. AF can lead to hemodynamic changes, abnormal atrioventricular synchronization, and impairments in atrial, ventricular, and ventricular mechanical functions. Severe cases will contribute to heart failure and sudden cardiac death, dramatically heightening mortality rates.^[13,14]^ Moreover, recurrent AF episodes sharply augment the risk of stroke, culminating in cognitive decline and vascular dementia, seriously affecting patient prognosis.^[15,16]^ The latest studies have pointed out that AF management is multifaceted. Since the last century, the treatment paradigm for AF has gradually shifted. Treatment is not only focused on arrhythmia but also emphasizes comprehensive management and risk factor control, which has become a crucial pillar of AF management.^[17]^ Currently, antiarrhythmic drugs and RFA are the 2 most commonly adopted approaches for treating AF. Nonetheless, due to the complex pathogenesis of AF and unclear treatment targets, clinical treatments lack sufficient effectiveness and safety. The probability of recurrence following RFA is also relatively high.^[18]^ Therefore, identifying parameters with high sensitivity and specificity for forecasting AF recurrence and using them to prevent AF recurrence after RFA is a research hotspot for the current stage.

NT-proBNP and cTnI levels are inextricably associated with the functional status of the human heart, and their abnormal alterations can serve as important indicators of cardiac disease progression. Ragonese et al^[19]^ have found that patients who have experienced cardiovascular events have higher average concentrations of NT-proBNP compared to those who have not experienced such events. An NT-proBNP level of 91.55 pg/mL predicts cardiovascular events effectively, with an OR value of 19.06. Elevated serum NT-proBNP has been identified as an independent midterm predictor of fatal or nonfatal cardiovascular events in acromegaly patients. Shen et al^[20]^ have also discovered that NT-proBNP and cTnI levels on the day of admission for acute myocardial infarction are notably higher than those on the day before discharge or on the day of discharge, suggesting that serum NT-proBNP and cTnI levels are efficacious markers for assessing the condition of acute myocardial infarction patients. In addition to serum biomarkers, UCG also performs excellently in predicting cardiac diseases. LAD, LVDD, LVEF, and LVMI are the most prevalent parameters measured in UCG and are important predictors of ventricular remodeling and adverse outcomes.^[21–23]^ A study on children with cardiac insufficiency caused by ventricular preexcitation asynchrony undergoing catheter ablation has discovered that as per Cox regression analysis, smaller LVDD and higher LVEF before ablation are predictive factors for the recovery time of cardiac function impairment. The higher the LVDD and the lower the LVEF, the poorer the improvement in cardiac function after ablation.^[24]^ Ince et al.^[25]^ have reported that transthoracic echocardiography is an efficacious tool for identifying and monitoring severe sepsis and septic myocardial dysfunction. Abnormal alterations in LVDD can reflect cardiovascular abnormalities in septic cardiomyopathy and are potential negative prognostic factors. These studies collectively demonstrate the remarkable value of serum NT-proBNP, cTnI, and UCG in cardiovascular diseases.

Notably, the recurrence cohort included a higher proportion of patients with NYHA class IV heart failure compared with the nonrecurrence cohort. Because advanced NYHA class reflects more severe cardiac dysfunction, it could confound the observed associations: patients with worse functional status may exhibit higher NT-proBNP and cTnI levels and larger chamber dimensions, as well as an intrinsically greater risk of postRFA recurrence. To mitigate this potential bias, we included NYHA class as a covariate (entered as dummy variables) in the multivariate logistic regression model, and variance inflation factors confirmed no problematic collinearity. Despite this adjustment, residual confounding may remain, potentially exaggerating the magnitude of associations between biomarkers, echocardiographic parameters, and recurrence risk. Therefore, our results should be interpreted with caution. Future studies employing propensity score matching or stratified analyses by NYHA functional class are warranted to isolate the independent predictive value of serological and imaging markers.

Here, we discovered that compared to the nonrecurrence cohort, patients with AF recurrence after RFA had dramatically heightened levels of serum NT-proBNP and cTnI, elevated LAD and LVDD, and lowered LVEF (*P* < .05). Logistic regression analysis denoted that serum markers NT-proBNP and cTnI, along with UCG parameters LAD, LVDD, and LVEF, were all independent influencing factors for AF recurrence postablation (*P* < .05). ROC curve outcomes also demonstrated that the highest efficacy in predicting AF recurrence postablation was achieved when NT-proBNP surpassed 807.17 pg/mL, cTnI was above 0.3 ng/mL, LAD reached above 34.99 mm, LVDD was above 43.14 mm, and LVEF was ≤ 59.15%. The AUC for the combined prediction of AF recurrence attained 0.771, with a sensitivity of 74.07% and specificity of 75.71%. Combined detection boasted a higher value in forecasting AF recurrence following surgery.

Similar to previous research, our study aligns with findings from Miao et al,^[26]^ who have unraveled that heart failure patients have poorer left heart structure and function, higher heart-type fatty acid-binding protein and cTnI levels compared to healthy controls. Logistic regression analysis has unveiled that heart-type fatty acid-binding protein and cTnI are risk factors for heart failure, whereas LVEF stands as a protective factor. Salem et al.^[27]^ have demonstrated that gauging BNP, MR-ProADM, and cTnI levels upon admission boasts high diagnostic value for disease progression and clinical severity of pediatric heart failure. BNP demonstrates a particular advantage in predicting prognosis in pediatric patients. Compared to healthy children, pediatric patients exhibit substantially heightened levels of all 3 biomarkers, and these levels are linearly correlated with the Ross score, ejection fraction, and length of hospital stay. Upon admission, brain natriuretic peptide predicts hospital mortality with a sensitivity of 95.5% and specificity of 88%. These studies collectively highlight the value of serum markers NT-proBNP, cTnI, and UCG parameters in forecasting cardiovascular disease and cardiac functional status. Our study not only analyzed the value of single-item detection in AF recurrence but also further explored the diagnostic efficacy of combined monitoring involving both serum markers and UCG parameters in forecasting AF recurrence postRFA. This provides robust predictive indicators and focused monitoring directions for prognosis assessment and disease progression in AF patients undergoing RFA.

Our findings demonstrate that the combined assessment of serological and echocardiographic markers offers significant discriminatory power for predicting AF recurrence after RFA. To translate this into clinical practice, future work should focus on embedding these predictors into real-time clinical decision support systems. For example, integrating biomarker levels with echocardiographic parameters could generate individualized recurrence risk scores, thereby informing tailored follow-up, anticoagulation strategies, or early reablation decisions. Importantly, decision curve analysis showed that our model provides greater net clinical benefit than either “treat all” or “treat none” approaches across a clinically relevant range of risk thresholds (10%–40%), reinforcing its potential utility in guiding patient-specific management. Further cost-effectivenessanalyses will be necessary to determine whether the added resource use associated with biomarker testing and imaging is justified by downstream reductions in recurrence, hospitalization, and long-term morbidity.

This study has several limitations. Although the sample size (n = 97) was modest, post hoc power analysis demonstrated sufficient statistical power to detect meaningful differences for all key predictors, particularly NT-proBNP, cTnI, and LVEF. However, the limited cohort size may still restrict the precision of effect estimates and the generalizability of findings. The 1-year follow-up period may not fully capture late arrhythmia recurrences or long-term prognostic implications. In addition, the single-center design limits external validity, and we did not quantify coronary artery disease burden, which may confound biomarker and imaging results. Future prospective studies should incorporate standardized coronary artery disease assessment and validate these findings in larger, multicenter populations with extended follow-up durations. While we adhered strictly to Strengthening the Reporting of Observational Studies in Epidemiology guidelines and applied blinded data collection with robust statistical methodology to ensure internal validity, further research is needed to refine the predictive model and confirm its clinical applicability across diverse settings.

## 5. Conclusion

In summation, serum markers NT-proBNP, cTnI, and UCG indices LAD, LVDD, and LVEF are independent factors that can influence AF recurrence subsequent to RFA. The combined detection method with all these markers incorporated boasts high value in predicting AF recurrence following RFA, which is worthy of clinical reference.

## Author contributions

**Conceptualization:** Jia-Fa Jin, Kun Feng, Xiu-Yu Wang.

**Data curation:** Jia-Fa Jin, Shen Huang, Kun Feng, Xiu-Yu Wang.

**Formal analysis:** Jia-Fa Jin.

**Investigation:** Jia-Fa Jin, Shen Huang, Xiu-Yu Wang.

**Methodology:** Jia-Fa Jin, Shen Huang, Kun Feng.

**Resources:** Jia-Fa Jin, Shen Huang, Kun Feng, Xiu-Yu Wang.

**Software:** Shen Huang, Kun Feng.

**Supervision:** Jia-Fa Jin.

**Writing – original draft:** Jia-Fa Jin.

**Writing – review & editing:** Jia-Fa Jin.

## Supplementary Material
