# Supplementary material for: Study on the application of serological indicators combined with ultrasonic cardiogram in predicting recurrence of radiofrequency ablation in atrial fibrillation patients
Source: Medicine (Baltimore). 2026 Mar 13;105(11):e47014. doi: 10.1097/MD.0000000000047014 (PMC12991452; doi:10.1097/MD.0000000000047014)
Supplement: Supplementary file 1 [file medi-105-e47014-s001.docx]

**Supplementary Tables**

**Supplementary Table 1.** Sensitivity–specificity trade-off at different cutoff values

| Variable | Cutoff Value | Sensitivity (%) | Specificity (%) |
| --- | --- | --- | --- |
| NT-proBNP | 750 pg/ml | 85.2 | 65.7 |
|  | 807.17 pg/ml (Youden) | 70.4 | 78.6 |
|  | 850 pg/ml | 63 | 82.9 |
| cTnI | 0.25 ng/ml | 81.5 | 66 |
|  | 0.30 ng/ml (Youden) | 66.7 | 80 |
|  | 0.35 ng/ml | 55.6 | 85.7 |
| LAD | 33 mm | 81.5 | 61.4 |
|  | 34.99 mm (Youden) | 70.4 | 70 |
|  | 37 mm | 55.6 | 77.1 |
| LVDD | 42 mm | 74.1 | 61.4 |
|  | 43.14 mm (Youden) | 48.1 | 85.7 |
|  | 45 mm | 40.7 | 90 |
| LVEF | 61% | 77.8 | 62.9 |
|  | 59.15% (Youden) | 59.3 | 75.7 |
|  | 57% | 48.1 | 82.9 |

Note: Optimal cutoffs determined by the Youden index are shown in bold. Alternative thresholds illustrate sensitivity–specificity trade-offs for clinical application.

**Supplementary Table 2.** Missing data by variable

| Variable | Type | Missing cases (n) | Missing proportion (%) |
| --- | --- | --- | --- |
| Age | Demographic | 0 | 0 |
| Sex | Demographic | 0 | 0 |
| Body Mass Index (BMI) | Demographic | 1 | 1 |
| Hypertension | Comorbidity | 0 | 0 |
| Diabetes | Comorbidity | 0 | 0 |
| Cerebrovascular disease | Comorbidity | 0 | 0 |
| AF type | Clinical | 0 | 0 |
| NYHA functional class | Clinical | 1 | 1 |
| NT-proBNP (pg/mL) | Serological | 3 | 3.1 |
| cTnI (ng/mL) | Serological | 2 | 2.1 |
| LAD (mm) | Echocardiographic | 4 | 4.1 |
| LVDD (mm) | Echocardiographic | 3 | 3.1 |
| LVEF (%) | Echocardiographic | 2 | 2.1 |
| LVMI (g/m²) | Echocardiographic | 3 | 3.1 |

Note: Overall missingness ranged from 0.0% to 4.3%. Little’s MCAR test indicated data were missing completely at random (P = 0.291). Variables with <5% missingness were imputed using MICE, and those with <1% missingness were handled by listwise deletion.
